# Survival outcomes of different neoadjuvant treatment regimens in patients with locally advanced rectal cancer and MRI‐detected extramural venous invasion

**DOI:** 10.1002/cam4.6625

**Published:** 2023-10-21

**Authors:** Mo Chen, Yan Ma, Yi‐wen Song, Jinhua Huang, Yuan‐hong Gao, Jian Zheng, Fang He

**Affiliations:** ^1^ Department of Genitourinary Oncology The First People's Hospital of Foshan Foshan Guangdong China; ^2^ Department of Radiation Oncology, Guangdong Provincial Key Laboratory of Colorectal and Pelvic Floor Diseases The Sixth Affiliated Hospital, Sun Yat‐sen University Guangzhou Guangdong China; ^3^ Biomedical Innovation Center, The Sixth Affiliated Hospital Sun Yat‐sen University Guangzhou Guangdong China; ^4^ Department of Minimal Invasive Interventional Therapy Sun Yat‐sen University Cancer Centre, State Key Laboratory of Oncology in South China, Collaborative Innovation Centre for Cancer Medicine Guangzhou Guangdong China; ^5^ Department of Radiation Oncology Sun Yat‐sen University Cancer Centre, State Key Laboratory of Oncology in South China, Collaborative Innovation Centre for Cancer Medicine Guangzhou Guangdong China

**Keywords:** extramural venous invasion, neoadjuvant chemotherapy, radiotherapy, rectal cancer, survival

## Abstract

**Purpose:**

MRI‐detected extramural venous invasion (mrEMVI) is associated with poor survival outcomes in patients with locally advanced rectal cancer (LARC). An mrEMVI‐positive status is considered a strong indication for neoadjuvant treatment, but the optimal regimen is unknown.

**Patients and Methods:**

We retrospectively compared pathological and survival outcomes of 584 patients diagnosed with mrEMVI‐positive rectal cancer between January 2013 and October 2021, and receiving either neoadjuvant chemotherapy (NCT) alone, neoadjuvant chemoradiotherapy (nCRT) alone, or nCRT plus NCT, prior to total mesorectal excision. Propensity score matching (PSM) was used to balance clinical bias between groups, which were compared using chi‐square testing and Kaplan–Meier curves.

**Results:**

Median follow‐up was 33.9 (range, 10.2–100.4) months. The 3‐year overall survival (OS), disease‐free survival (DFS), distant metastasis‐free survival (DMFS), and locoregional relapse‐free survival (LRFS) rates for all patients were 90.4%, 57.5%, 61.1%, and 85.7%, respectively. Of 584 mrEMVI‐positive patients at the time of diagnosis, 457 (78.3%) were EMVI‐negative on surgical pathology, and they had significantly better 3‐year OS, DMFS, DFS, and LRFS rates (all *p* < 0.001) than patients who remained EMVI‐positive. After PSM was applied, patients receiving nCRT alone had significantly better 3‐year OS (96.8% vs. 86.5%, *p* = 0.005) and DMFS (67.1% vs. 53.5%, *p* = 0.03) rates than those receiving NCT alone. Patients receiving NCT plus nCRT had higher pathological complete response (PCR) (10.8% vs. 2.7%, *p* = 0.04) and downstaging (33.8% vs. 5.3%, *p* < 0.001) rates than those receiving nCRT alone, but survival rates did not differ (all *p* > 0.05).

**Conclusion:**

Most EMVI‐positive patients with LARC converted to EMVI‐negative after neoadjuvant treatment, resulting in improved OS and DFS. Patients receiving nCRT had more favorable survival outcomes than those receiving NCT, suggesting the importance of including neoadjuvant radiotherapy. Patients receiving NCT in addition to nCRT had higher rates of PCR and downstaging, but their survival rates were not better.

## Graphical abstract

Patients with MRI‐detected EMVI at the time of their rectal cancer diagnosis have exhibited significantly worse survival outcomes, which is now considered an important prognostic factor for rectal cancer. Patients who received nCRT had more favorable survival outcomes than those who received NCT only before TME surgery, suggesting that neoadjuvant radiotherapy was indispensable in EMVI‐positive LARC patients.

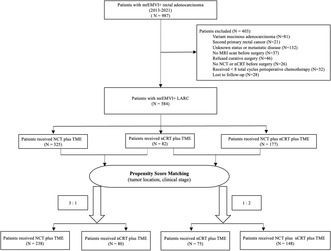

## 1 INTRODUCTION

For patients with rectal cancer, extramural venous invasion (EMVI) is associated with high rates of distant metastasis and low rates of overall survival (OS).^1^, ^2^ Traditionally, EMVI has been diagnosed by the pathological analysis of surgical tumor specimens. However, accurate detection of EMVI on pathologic examination (pEMVI) has been hampered by sampling bias and the heterogeneity of definitions and evaluation methodologies used by pathologists. In recent years, advances in high‐quality magnetic resonance imaging (MRI) have resulted in more accurate staging and improvements in the identification of pathological findings, including EMVI.^3^, ^4^, ^5^

Because patients with MRI‐detected EMVI (mrEMVI) at the time of their rectal cancer diagnosis have exhibited significantly worse survival outcomes, this is now considered a useful prognostic factor for rectal cancer.^6^ The European Society for Medical Oncology (ESMO) guidelines for rectal cancer now recommend that mrEMVI status be determined to help guide treatment decisions and that an mrEMVI‐positive status be considered a strong indication for neoadjuvant treatment.^7^

Neoadjuvant chemotherapy (NCT) has been used for years in patients with locally advanced rectal cancer (LARC), who are known to have a substantial risk of distant recurrence. In addition, neoadjuvant chemoradiotherapy (nCRT) is now commonly offered to these patients.^8^ Furthermore, intensification of neoadjuvant treatment with induction or consolidation chemotherapy before surgery, an approach known as total neoadjuvant treatment (TNT),^9^ has been shown to significantly decrease the probability of disease‐related systemic treatment failure in patients with LARC.^10^, ^11^

However, the need for radiotherapy in patients with LARC remains controversial,^12^ particularly for those without adverse prognostic indicators. Neoadjuvant radiotherapy contributes to excellent local control in patients with rectal cancer, but it does not impact distant recurrence or OS rates.^13^ Meanwhile, it is associated with additional morbidity, increased surgical complications, and a poorer quality of life in these patients.^14^ Furthermore, in the FOWARC study of patients with LARC, treatment with NCT alone resulted in comparable survival to treatment with nCRT alone or nCRT plus NCT.^15^ And, in the CONVERT study, NCT alone followed by surgery was found to be an effective alternative to nCRT for patients with LARC and uninvolved mesorectal fascia (MRF).^16^

Despite reports of its usefulness, mrEMVI status has not been routinely reported or analyzed in many of the published studies involving patients with LARC. Yet, there is evidence that when patients receive nCRT alone and an mrEMVI‐positive status is converted to an mrEMVI‐negative status, the negative impact of EMVI on survival may be eliminated.^17^ On the other hand, although NCT alone results in an mrEMVI conversion rate as high as 80%, significant improvements in survival have not followed.^18^ Thus, trials involving patients with LARC who are stratified by mrEMVI status are needed to help determine the optimal neoadjuvant treatment regimen for patients with this adverse prognostic indicator.

To explore this issue, we conducted a study with propensity score matching (PSM) that involved patients with LARC who were mrEMVI‐positive at the time of diagnosis and who received various forms of neoadjuvant treatment followed by total mesorectal excision (TME) at our hospital. Our goal was to determine the pathological and survival outcomes of patient with LARC who were treated with either NCT alone, nCRT alone, or nCRT plus NCT, all prior to curative surgery.

## 2 PATIENTS AND METHODS

### 2.1 Patient selection

Our Review Board approved this retrospective study. A database search at our institution revealed 5126 patients who had been diagnosed with and treated for rectal cancer between January 2013 and October 2021. Of these, 987 (19.3%) patients were mrEMVI‐positive on their baseline MRI.

**FIGURE 1:**
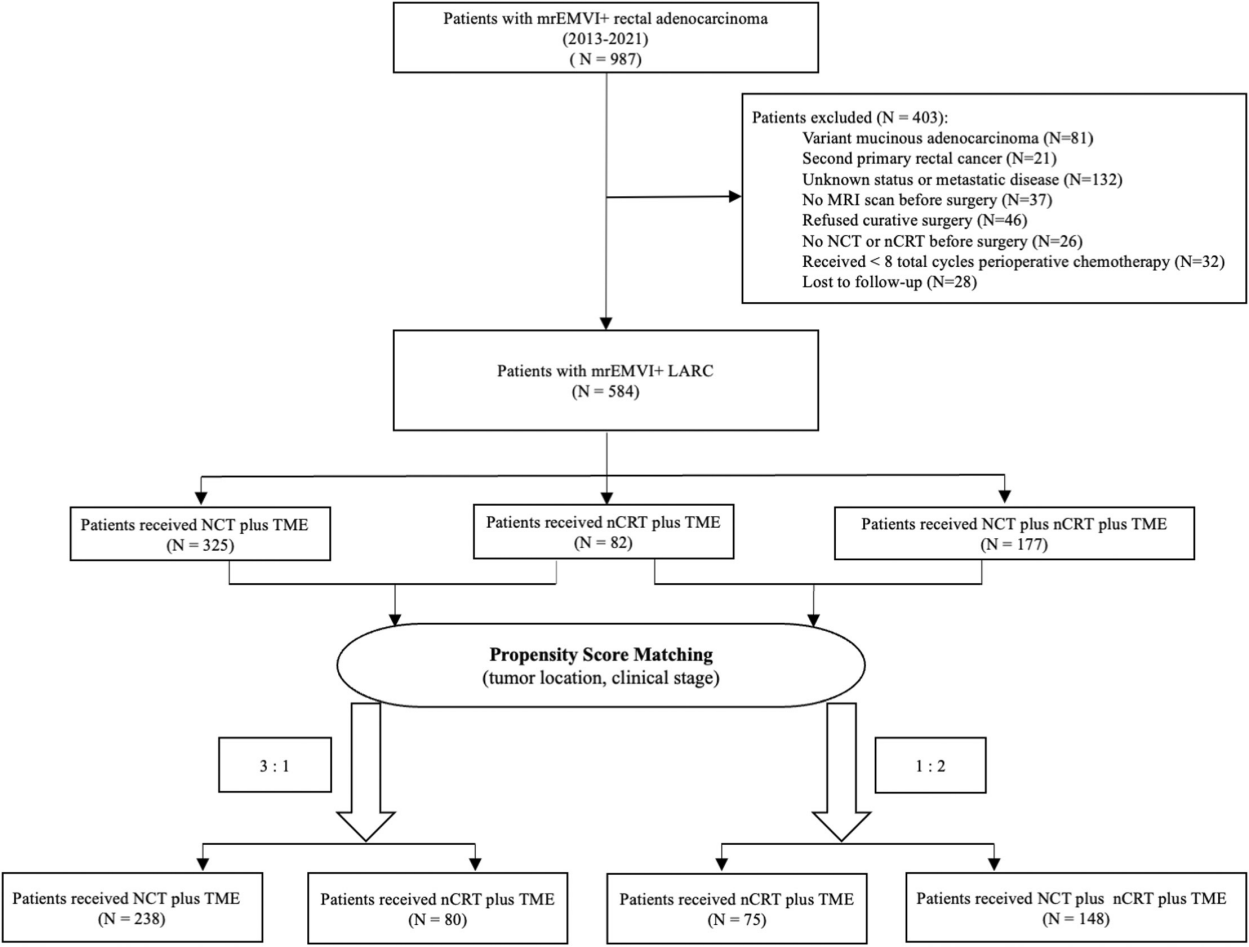
CONSORT flow diagram. LARC, locally advanced rectal cancer; mrEMVI, MRI‐detected extramural venous invasion; MRI, magnetic resonance imaging; NCT, neoadjuvant chemotherapy; nCRT, neoadjuvant chemoradiotherapy; TME, total mesorectal excision.

At last, 584 (59.2%) met the following inclusion criteria: (a) had biopsy‐proven rectal adenocarcinoma, (b) had no distant metastases, (c) intended at the time of diagnosis to pursue curative treatment, and (d) had TME, and (e) had received NCT and/or nCRT prior to TME (Figure 1). A total of 403 patients were excluded from the study because they had other histologic type instead of adenocarcinoma (i.e., variant mucinous adenocarcinoma), second primary rectal cancer, unknown status or metastatic disease, no MRI scan before surgery, refused curative surgery (i.e., chose palliative therapy), received no NCT or nCRT before surgery, received less than eight total cycles of perioperative chemotherapy, or were lost to follow‐up.

### 2.2 MRI analysis

All MRI scans were retrospectively blindly reviewed for mrEMVI status, tumor location from anal verge, MRF status, and lateral lymph node status by one of two gastrointestinal radiologists specializing in abdominal radiology. When there were any inconsistencies between the opinion of the radiologist and the original results, they review the images until reach a consensus. For each patient, high‐resolution T2WI, CE‐TIWI, and DWI with two b values (0 and 800 or 1000 s/mm^2^) were acquired by 1.5 or 3.0 T scanners in our hospital. The diagnosis of EMVI on MRI was based primarily on axial and coronal T2WI, but T1WI, additional DWI and post‐gadolinium images were also consulted.

### 2.3 Histopathology evaluation

The whole specimen after curative surgery was reported by the specialist gastrointestinal histopathologist. The diagnosis of EMVI was primarily on hematoxylin–eosin stain. At our institution, Elastic‐Van Gieson was not routinely used to assess EMVI status, except as an aid to diagnosis.

### 2.4 Neoadjuvant and adjuvant treatment

All patients in the study received neoadjuvant treatment and at least eight total cycles of perioperative chemotherapy. Patients had either NCT alone, nCRT alone, or nCRT plus NCT.

Based on the opinion of the multidisciplinary team, a portion of the patients received adjuvant chemotherapy (ACT) after surgery. The chemotherapy regimens used consisted of a fluoropyrimidine‐based regimen. All patients had nCRT that involved intensity‐modulated radiotherapy (IMRT). The chemotherapy and radiotherapy regimens were performed using similar method that had been previously described.^19^

### 2.5 Follow‐up and outcomes

The duration of follow‐up was defined as the time from the first day of any treatment to either the date of last examination or the date of death. Patients were routinely assessed at 3‐month intervals during the first 2 years of care and at 6‐month intervals thereafter. The median follow‐up for the entire cohort was 33.9 (range, 10.2–100.4) months.

Primary outcomes used in this study included OS, disease‐free survival (DFS), distant metastasis‐free survival (DMFS) and locoregional relapse‐free survival (LRFS). OS was defined as the interval from surgery to death as a result of any cause; DFS was defined as the interval from surgery to the first disease relapse at any site or death; DMFS, defined by time from the initiation of treatment to the first distant (i.e., outside the pelvis) relapse or death; and LRFS, defined by time from the initiation of treatment to the first locoregional (i.e., within the pelvis) relapse or death.

Secondary outcomes included EMVI status, MRF status, pathological complete response (PCR), downstaging, and circumferential resection margin (CRM) status. For this study, the conversion of a patient from EMVI‐positive to EMVI‐negative status was defined as having a pre‐treatment MRI showing EMVI and a surgical pathology report showing no EMVI (i.e., no vascular invasion). MRI‐detected MRF (mrMRF) involvement was defined as being either negative (>1 mm) or positive (≤1 mm). A PCR was defined as the absence of any viable tumor cells or lymph‐node metastases in the resected specimens (i.e., stage ypT0N0). Downstaging of a patient was defined as having a pretreatment clinical stage II or III and a postoperative stage of ypT0‐2N0.

### 2.6 Study design and propensity score matching

Patients were divided into three groups: (1) the NCT alone group, who received NCT only; (2) the nCRT alone group, who received concurrent chemoradiotherapy only; and (3) the nCRT plus NCT group, who received both concurrent chemoradiotherapy and NCT (before or after nCRT, but prior to surgery). To minimize the bias of confounding factors, PSM analysis was conducted with the goal of facilitating comparability between these groups. For this process, we used clinical TNM stage and distal tumor distance from the anal verge. Using these criteria, we obtained matching of propensity scores with a 3:1 optimal matching method between the NCT alone and nCRT alone groups, and with a 2:1 optimal matching method between the nCRT alone and nCRT plus NCT groups.

### 2.7 Statistical methods

The chi‐squared (*χ* ^2^) test was used to compare the distributions of the demographic and clinicopathological characteristics between groups. Kaplan–Meier survival curves were used to compare patient survival outcomes between groups. Statistical differences between curves were calculated using the log‐rank test. All survival outcome measures were censored on October 1, 2022. All *p* values were two‐sided, and a *p* value less than 0.05 was considered statistically significant. Statistical analyses were performed with SPSS (version 24.0; SPSS, Inc., Chicago, IL).

## 3 RESULTS

### 3.1 Study population characteristics prior to propensity score matching

Baseline patient characteristics are shown in Table 1. Of the 584 study patients who had LARC, were mrEMVI‐positive, and underwent TME, 325 (55.7%) received NCT alone, 82 (14.0%) received nCRT alone, and 177 (30.3%) received NCT plus nCRT.

**TABLE 1:** Demographic and clinicopathological characteristics of 584 patients with locally advanced rectal cancer (LARC) and MRI‐detected extramural venous invasion (mrEMVI), by type of treatment, January 2013 through October 2021.

Characteristics	Patients *n* (%) (*N* = 584)	NCT alone *n* (%) (*N* = 325)	nCRT alone *n* (%) (*N* = 82)	NCT + nCRT *n* (%) (*N* = 177)	*p* value
Age^a^, years					**0.03**
≤60	306 (52.4)	155 (47.7)	51 (62.2)	100 (56.5)	
>60	278 (47.6)	170 (52.3)	31 (37.8)	77 (43.5)	
Gender					0.41
Male	434 (74.3)	236 (72.6)	60 (73.2)	138 (78.0)	
Female	150 (25.7)	89 (27.4)	22 (26.8)	39 (22.0)	
Before neoadjuvant treatment					
Clinical T stage					0.16
cT2	1 (0.2)	1 (0.3)	0 (0.0)	0 (0.0)	
cT3	306 (52.4)	178 (54.8)	48 (58.5)	80 (45.2)	
cT4	277 (47.4)	146 (44.9)	34 (41.5)	97 (54.8)	
Clinical *N* stage					**0.03**
cN0	42 (7.2)	32 (9.8)	4 (4.9)	6 (3.4)	
cN1	185 (31.7)	108 (33.2)	21 (25.6)	56 (31.6)	
cN2	357 (61.1)	185 (56.9)	57 (69.5)	115 (65.0)	
Clinical stage					**0.009**
II	41 (7.0)	32 (9.8)	4 (4.9)	5 (2.8)	
III	543 (93.0)	293 (90.2)	78 (95.1)	172 (97.2)	
Tumor differentiation (initial biopsy)					0.89
High	54 (9.2)	32 (9.8)	6 (7.3)	16 (9.0)	
Moderate	465 (79.6)	255 (78.5)	66 (80.5)	144 (81.4)	
Poor	65 (11.2)	38 (11.7)	10 (12.2)	17 (9.6)	
MRI‐detected tumor location from anal verge, cm					**<0.001**
<5	155 (26.5)	65 (20.0)	23 (28.0)	67 (37.9)	
5–10	318 (54.5)	170 (52.3)	49 (59.8)	99 (55.9)	
>10	111 (19.0)	90 (27.7)	10 (12.2)	11 (6.2)	
mrMRF status					**0.04**
Negative (>1 mm)	93 (15.9)	60 (18.5)	15 (18.3)	18 (10.2)	
Positive (≤1 mm)	491 (84.1)	265 (81.5)	67 (81.7)	159 (89.8)	
MRI‐detected lateral lymph node status					**0.003**
Negative	429 (73.5)	255 (78.5)	60 (73.2)	114 (64.4)	
Positive	155 (26.5)	70 (21.5)	22 (26.8)	63 (35.6)	
After neoadjuvant treatment, before surgery					
mrEMVI status					**0.89**
Negative	513 (87.8)	286 (88.0)	73 (89.0)	154 (87.0)	
Positive	71 (12.2)	39 (12.0)	9 (11.0)	23 (13.0)	
mrMRF status					**0.46**
Negative	398 (68.2)	217 (66.7)	54 (65.9)	127 (71.8)	
Positive	186 (31.8)	108 (33.2)	28 (34.1)	50 (28.2)	
After surgery					
Pathological complete response (PCR)					**0.001**
Yes	30 (5.1)	10 (3.1)	2 (2.4)	18 (10.2)	
No	554 (94.9)	315 (96.9)	80 (97.6)	159 (89.8)	
Pathological T stage					**<0.001**
ypT0	35 (6.0)	12 (3.7)	2 (2.4)	21 (11.9)	
ypT1–T2	108 (18.5)	56 (17.2)	5 (6.1)	47 (26.6)	
ypT3–T4	441 (75.5)	257 (79.1)	75 (91.5)	109 (61.6)	
Pathological *N* stage					**<0.001**
ypN0	291 (49.8)	147 (45.2)	30 (36.6)	114 (64.4)	
ypN1	204 (34.9)	120 (36.9)	36 (43.9)	48 (27.1)	
ypN2	89 (15.3)	58 (17.9)	16 (19.5)	15 (8.5)	
Downstaging (ypT0–2N0)^b^					**<0.001**
Yes	105 (18.0)	43 (13.2)	4 (4.9)	58 (32.8)	
No	479 (82.0)	282 (86.8)	78 (95.1)	119 (67.2)	
ypEMVI status					**0.001**
Negative	457 (78.3)	239 (73.5)	62 (75.6)	156 (88.1)	
Positive	127 (21.7)	86 (26.5)	20 (24.4)	21 (11.9)	
Neural invasion					**0.005**
Negative	440 (75.3)	237 (72.9)	55 (67.1)	148 (83.6)	
Positive	144 (24.7)	88 (27.1)	27 (32.9)	29 (16.4)	
CRM status					**0.02**
Negative (>1 mm)	573 (98.1)	318 (97.8)	78 (95.1)	177 (100.0)	
Positive (≤1 mm)	11 (1.9)	7 (2.2)	4 (4.9)	0 (0.0)	

	Median (IQR)	Median (IQR)	Median (IQR)	Median (IQR)	*p* value
Time start treatment to surgery, month	5.1 (4.1–6.7)	4.3 (3.5–5.1)	5.1 (4.8–5.8)	7.1 (6.5–7.7)	—
Total chemotherapy cycles	12 (10–12)	12 (10–12)	12 (11–12)	12 (10–12)	—

*Note*: The numbers in bold are all numbers with a *p*‐value < 0.05. In order to show that these values have statistical differences. We believe that it is acceptable for numbers not to be in bold.

Abbreviations: CRM, circumferential resection margin; IQR, interquartile range; mo, months; mrEMVI, MRI‐detected extramural venous invasion; MRI, magnetic resonance imaging; mrMRF, MRI‐detected mesorectal fascia; nCRT, neoadjuvant chemoradiotherapy; NCT, neoadjuvant chemotherapy; ypEMVI, pathological EMVI after neoadjuvant therapy and surgery.

^a^ Median (IQR) age 60 (50–67) years.

^b^ Downstaging defined as having a pretreatment clinical stage II or III and a postoperative stage of ypT0‐2N0.

Compared with patients in the other two groups, more patients in the NCT alone group were over 60 years old (*p* = 0.03), have a less advanced clinical *N* stage (i.e., cN0 or cN1) (*p* = 0.03), have a less advanced clinical stage (i.e., stage II) (*p* = 0.009), and have a tumor located in the upper rectum (i.e., >10 cm from the anal verge) (*p* < 0.001) (Table 1).

Compared with patients in the other two groups, more patients in the NCT plus nCRT group were mrMRF‐positive (*p* = 0.04) and MRI‐detected lateral lymph node (LLN) metastasis‐positive (*p* = 0.003) (Table 1). On the other hand, patients in the NCT plus nCRT group were also significantly more likely after surgery to demonstrate a PCR (*p* = 0.001) and downstaging (i.e., to pT0–T2 or pN0) (Both *p* < 0.001). Also, patients in the NCT plus nCRT group were significantly more likely on postoperative pathology to have negative EMVI (*p* = 0.001), neural invasion (*p* = 0.005), and CRM (*p* = 0.02). Of additional note, the NCT plus nCRT group had a longer median time between the start of treatment and surgery (7.1 months) than the nCRT alone group (5.1 months) and the NCT alone group (4.3 months).

### 3.2 Survival outcomes prior to propensity score matching

The 3‐year DFS, DMFS, LRFS, and OS rates for the entire cohort of patients were 57.5%, 61.1%, 85.7%, and 90.4%, respectively (Table 2). Compared to the other two groups, the NCT alone group had significantly lower DMFS (56.7%, *p* = 0.01) and OS (86.5%, *p* = 0.009) rates. The NCT alone group also had a lower 3‐year DFS rate (53.1%) than the nCRT alone (61.1%) and NCT plus nCRT (64.7%) groups, but these differences were not statistically significant (*p* = 0.13).

**TABLE 2:** Survival of 584 patients with locally advanced rectal cancer (LARC) and MRI‐detected extramural venous invasion (mrEMVI), by type of treatment, January 2013 through October 2021.

Survival	Patients (*N* = 584)	NCT alone (*N* = 325)	nCRT alone (*N* = 82)	NCT + nCRT (*N* = 177)	*p* value
3‐year DFS	57.5%	53.1%	61.1%	64.7%	0.13
3‐year DMFS	61.1%	56.7%	64.8%	67.7%	**0.01**
3‐year LRFS	85.7%	85.5%	87.2%	85.4%	0.65
3‐year OS	90.4%	86.5%	98.6%	94.3%	**0.009**

*Note*: The numbers in bold are all numbers with a *p*‐value < 0.05. In order to show that these values have statistical differences. We believe that it is acceptable for numbers not to be in bold.

Abbreviations: DFS, disease‐free survival; DMFS, distant metastasis‐free survival; LRFS, locoregional relapse‐free survival; nCRT, neoadjuvant chemoradiotherapy; NCT, neoadjuvant chemotherapy; OS, overall survival.

### 3.3 Survival outcomes of EMVI‐negative versus EMVI‐positive groups

**FIGURE 2:**
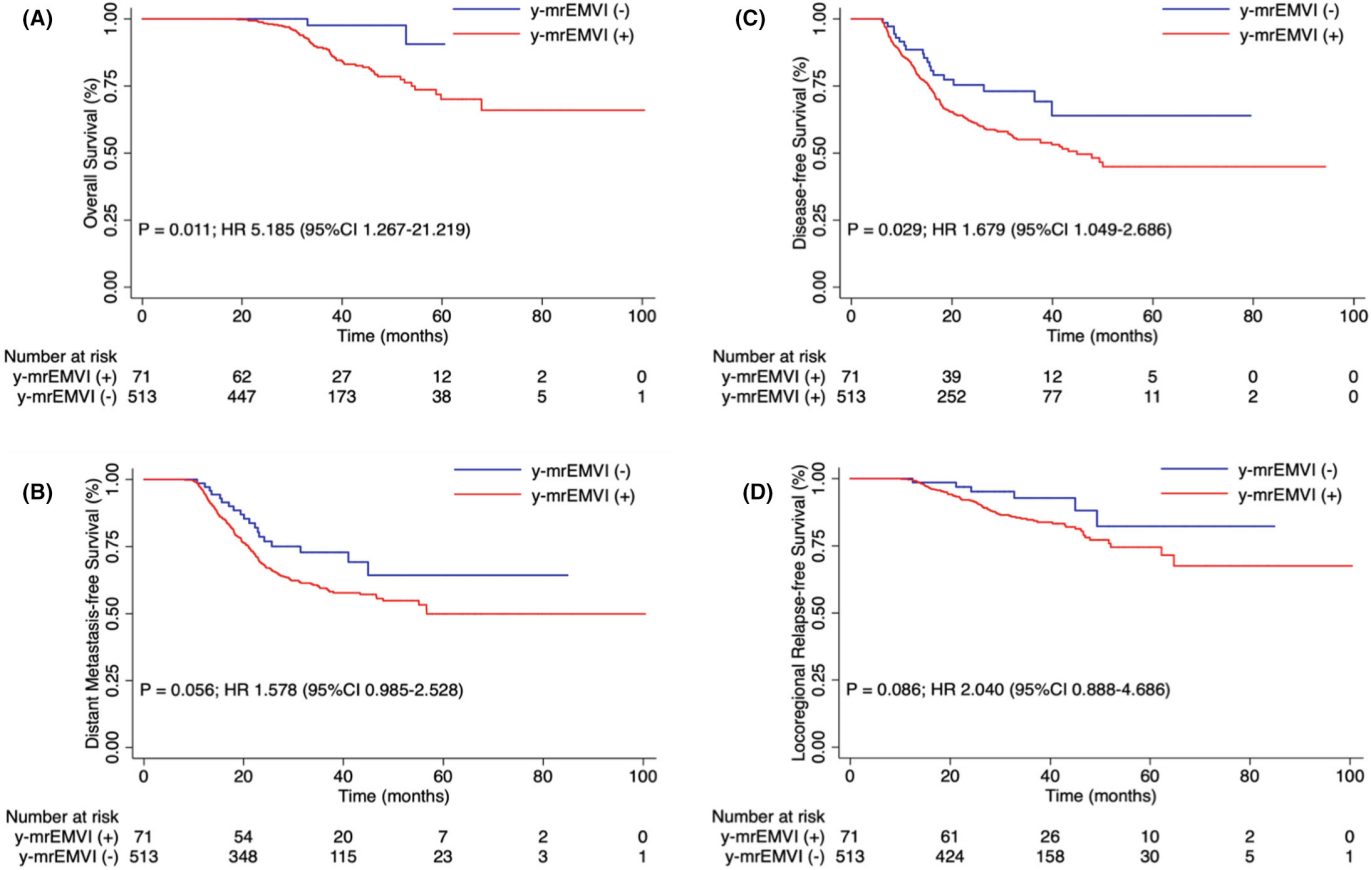
Kaplan–Meier curve analyses of 584 patients with magnetic resonance imaging (MRI)‐detected extramural venous invasion (EMVI)‐positive locally advanced rectal cancer (LARC), comparing those who were found on MRI to be EMVI‐negative (*N* = 513) to those who were found to be EMVI‐positive (*N* = 71), after neoadjuvant therapy, for (A) overall survival, (B) distant metastasis‐free survival, (C) disease‐free survival, and (D) locoregional relapse‐free survival rates, January 2013 through October 2021.

**FIGURE 3:**
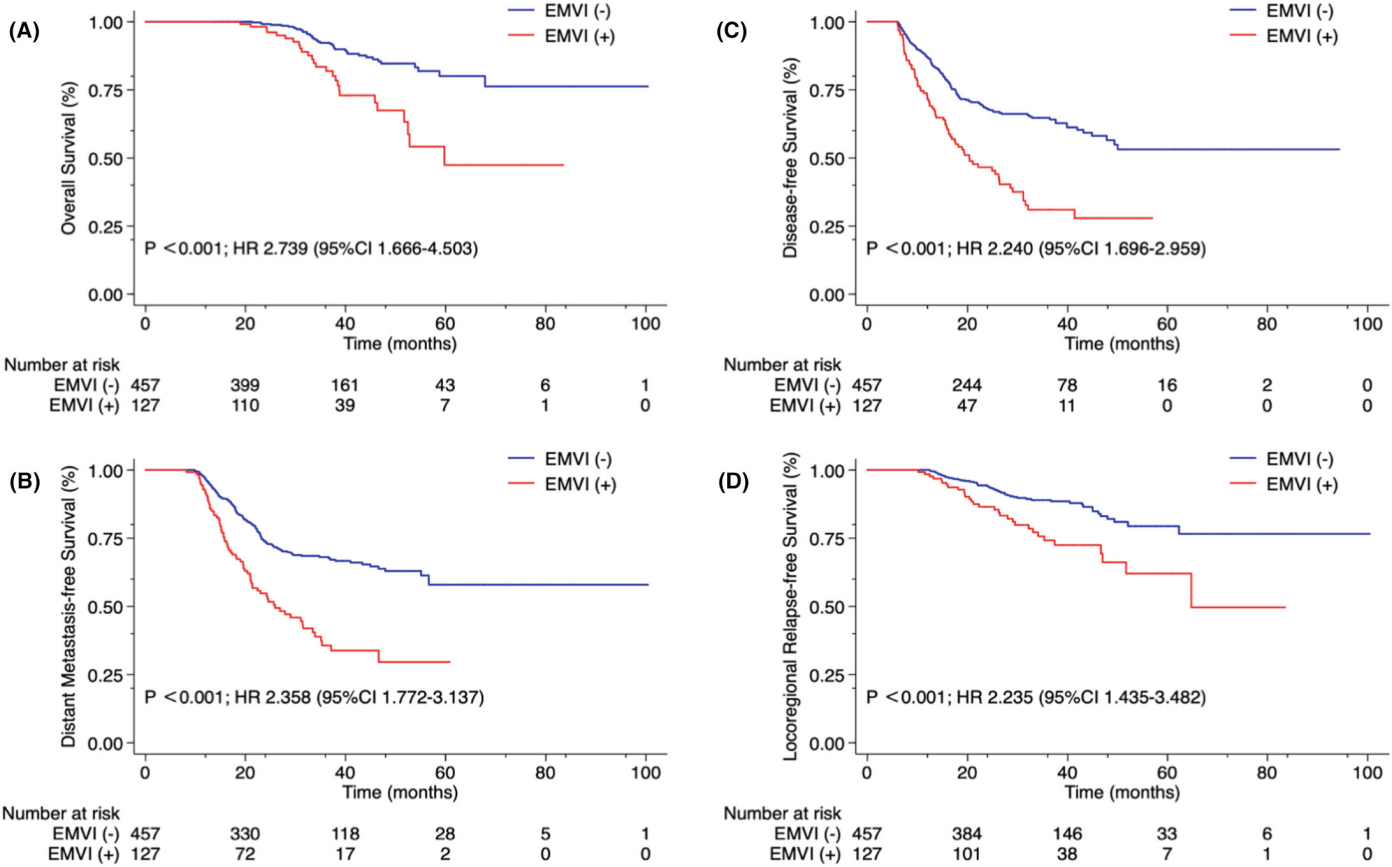
Kaplan–Meier curve analyses of 584 patients with magnetic resonance imaging (MRI)‐detected extramural venous invasion (EMVI)‐positive locally advanced rectal cancer (LARC), comparing those who were found on surgical pathology to be EMVI‐negative (*N* = 457) to those who were found to be EMVI‐positive (*N* = 127), after neoadjuvant therapy and surgery, for (A) overall survival, (B) distant metastasis‐free survival, (C) disease‐free survival, and (D) locoregional relapse‐free survival rates, January 2013 through October 2021.

Of the 584 patients who were mrEMVI‐positive at the time of diagnosis, 513 (87.8%) were mrEMVI‐negative after neoadjuvant treatment by MRI evaluation (y‐mrEMVI), and 457 (78.3%) were found on postoperative surgical pathology to have converted (based on the definition used for our study) to EMVI‐negative (Table 1). On Kaplan–Meier survival curves, patients who achieved y‐mrEMVI negative status demonstrated significantly better 3‐year OS (97.6% vs. 89.4%; *p* = 0.011) and 3‐year DFS (73.1% vs. 55.1%; *p* = 0.029) rates, and comparable 3‐year DMFS (72.8% vs. 59.4%; *p* = 0.056) and 3‐year LRFS (92.8% vs. 84.7%; *p* = 0.086) rates when compared to patients who remained y‐mrEMVI positive (Figure 2). On Kaplan–Meier survival curves, patients who achieved pathological EMVI‐negative status demonstrated significantly better 3‐year OS (92.3% vs. 83.4%; *p* < 0.001), 3‐year DMFS (68.1% vs. 35.7%; *p* < 0.001), 3‐year DFS (64.7% vs. 31.0%; *p* < 0.001), and 3‐year LRFS (89.0% vs. 74.2%; *p* < 0.001) rates when compared to patients who remained EMVI‐positive (Figure 3).

### 3.4 Study population characteristics after propensity score matching

#### 3.4.1 NCT alone group matched with nCRT alone group (3:1)

238 patients treated with NCT alone followed by TME were propensity score matched with 80 patients treated with nCRT alone followed by TME.

In total, 238 patients in NCT alone group were propensity score matched with 80 patients in nCRT alone group. Despite PSM, the NCT alone group still had a significantly higher proportion of patients than the nCRT alone group who were over 60 years old (53.4% vs. 38.8%, *p* = 0.03) and who had downstaging (13.9% vs. 5.0%, *p* = 0.04) (Table 3). However, no other significant differences between the two groups were observed (all *p* > 0.05).

**TABLE 3:** Demographic and clinicopathological characteristics of patients with locally advanced rectal cancer (LARC) and MRI‐detected extramural venous invasion (mrEMVI), by type of treatment, January 2013 through October 2021, after Propensity Score Matching.

Characteristics	After matching (3:1)		*p* value	After matching (1:2)		*p* value
	NCT alone *n* (%) (*N* = 238)	nCRT alone *n* (%) (*N* = 80)		nCRT alone n (%) (*N* = 75)	NCT + nCRT *n* (%) (*N* = 148)	
Age, years			**0.03**			0.67
≤60	111 (46.6)	49 (61.2)		46 (61.3)	86 (58.1)	
>60	127 (53.4)	31 (38.8)		29 (38.7)	62 (41.9)	
Gender			0.88			0.41
Male	178 (74.8)	59 (73.8)		55 (73.3)	116 (78.4)	
Female	60 (25.2)	21 (26.2)		20 (26.7)	32 (21.6)	
Before neoadjuvant treatment						
Clinical T stage			0.83			0.07
cT2	1 (0.4)	0 (0.0)		—	—	
cT3	142 (59.7)	47 (58.8)		45 (60.0)	69 (46.6)	
cT4	95 (39.9)	33 (41.3)		30 (40.0)	79 (53.4)	
Clinical *N* stage			0.94			0.57
cN0	10 (4.2)	4 (5.0)		2 (2.7)	5 (3.4)	
cN1	84 (35.3)	27 (33.8)		20 (26.7)	49 (33.1)	
cN2	144 (60.5)	49 (61.3)		53 (70.6)	94 (63.5)	
Clinical stage			0.99			0.99
II	9 (3.8)	3 (3.8)		2 (2.7)	4 (2.7)	
III	229 (96.2)	77 (96.3)		73 (97.3)	144 (97.3)	
Tumor differentiation (initial biopsy)			0.60			0.97
High	25 (10.5)	6 (7.5)		6 (8.0)	12 (8.1)	
Moderate	190 (79.8)	64 (80.0)		60 (80.0)	120 (81.1)	
Poor	23 (9.7)	10 (12.5)		9 (12.0)	16 (10.8)	
MRI‐detected tumor location from anal verge, cm			0.999			0.99
<5	62 (26.1)	21 (26.3)		21 (28.0)	42 (28.4)	
5–10	146 (61.3)	49 (61.3)		49 (65.3)	97 (65.5)	
>10	30 (12.6)	10 (12.5)		5 (6.7)	9 (6.1)	
mrMRF status			0.96			0.07
Negative (>1 mm)	44 (18.5)	15 (18.7)		15 (20.0)	16 (10.8)	
Positive (≤ 1 mm)	194 (81.5)	65 (81.3)		60 (80.0)	132 (89.2)	
MRI‐detected lateral lymph node status			0.54			0.36
Negative	184 (77.3)	59 (73.8)		55 (73.3)	99 (66.9)	
Positive	54 (22.7)	21 (26.3)		20 (26.7)	49 (33.1)	
After neoadjuvant treatment, before surgery						
mrEMVI status			0.46			0.52
Negative	201 (84.5)	71 (88.8)		68 (90.7)	128 (86.5)	
Positive	37 (15.5)	9 (11.2)		7 (9.3)	20 (13.5)	
mrMRF status			0.78			0.28
Negative (>1 mm)	160 (67.2)	52 (65.0)		48 (64.0)	106 (71.6)	
Positive (≤1 mm)	78 (32.8)	28 (35.0)		27 (36.0)	42 (28.4)	
After surgery						
Pathological complete response (PCR)			0.99			**0.04**
Yes	6 (2.5)	2 (2.5)		2 (2.7)	16 (10.8)	
No	232 (97.5)	78 (97.5)		73 (97.3)	132 (89.2)	
Pathological T stage			0.04			**<0.001**
ypT0	8 (3.3)	2 (2.5)		2 (2.7)	18 (12.1)	
ypT1	5 (2.1)	0 (0.0)		0 (0.0)	11 (7.4)	
ypT2	43 (18.1)	5 (6.3)		5 (6.7)	26 (17.6)	
ypT3	169 (71.0)	70 (87.5)		65 (86.6)	91 (61.5)	
ypT4	13 (5.5)	3 (3.8)		3 (4.0)	2 (1.4)	
Pathological N stage			0.47			**0.001**
ypN0	108 (45.3)	30 (37.5)		30 (40.0)	97 (65.5)	
ypN1	92 (38.7)	35 (43.8)		31 (41.3)	37 (25.0)	
ypN2	38 (16.0)	15 (18.7)		14 (18.7)	14 (9.5)	
Pathological TN stage			0.13			**<0.001**
ypT0N0	6 (2.5)	2 (2.5)		2 (2.7)	16 (10.8)	
ypI	27 (11.3)	2 (2.5)		2 (2.7)	34 (23.0)	
ypII	73 (30.7)	26 (32.5)		25 (33.3)	46 (31.1)	
ypIII	132 (55.5)	50 (62.5)		46 (61.3)	52 (35.1)	
Downstaging (ypT0‐2N0)^a^			**0.04**			**<0.001**
Yes	33 (13.9)	4 (5.0)		4 (5.3)	50 (33.8)	
No	205 (86.1)	76 (95.0)		71 (94.7)	98 (66.2)	
ypEMVI status			0.46			0.71
Negative	177 (74.4)	63 (78.8)		61 (81.3)	124 (83.8)	
Positive	61 (25.6)	17 (21.3)		14 (18.7)	24 (16.2)	
Neural invasion			0.19			0.07
Negative	179 (75.2)	54 (67.5)		51 (68.0)	118 (79.7)	
Positive	59 (24.8)	26 (32.5)		24 (32.0)	30 (20.3)	
CRM status			0.51			0.26
Negative (>1 mm)	230 (96.6)	76 (95.0)		73 (97.3)	147 (99.3)	
Positive (≤1 mm)	8 (3.4)	4 (5.0)		2 (2.7)	1 (0.7)	

	Median (IQR)	Median (IQR)	*p* value	Median (IQR)	Median (IQR)	*p* value
Time start treatment to surgery, months	4.3 (3.5–5.1)	5.1 (4.8–5.9)	0.66	5.1 (4.8–5.9)	7.1 (6.4–7.7)	**<0.001**
Total chemotherapy cycles	12 (10–12)	12 (11–12)	—	12 (11–12)	12 (10–12)	—
Preoperative chemotherapy cycles	6 (6–8)	4 (3–6)	—	4 (3–6)	8 (6–9)	—
ACT cycles	5 (0–6)	8 (6–8)	—	7 (6–8)	4 (0–6)	—

*Note*: The numbers in bold are all numbers with a *p*‐value < 0.05. In order to show that these values have statistical differences. We believe that it is acceptable for numbers not to be in bold.

Abbreviations: ACT, adjuvant chemotherapy; CRM, circumferential resection margin; IQR, interquartile range; mo, months; mrEMVI, MRI‐detected extramural venous invasion; MRI, magnetic resonance imaging; mrMRF, MRI‐detected mesorectal fascia; nCRT, neoadjuvant chemoradiotherapy; NCT, neoadjuvant chemotherapy; ypEMVI, pathological EMVI after neoadjuvant therapy and surgery.

^a^ Downstaging defined as having a pretreatment clinical stage II or III and a postoperative stage of ypT0‐2N0.

#### 3.4.2 nCRT alone group matched with NCT plus nCRT group (1:2)

A total of 75 patients in nCRT alone group were propensity score matched with 148 patients in NCT plus nCRT group. When compared with the nCRT alone group, the NCT plus nCRT group had a significantly longer interval between treatment initiation and surgery (7.1 months vs. 5.1 months, *p* < 0.001) (Table 3). Moreover, the NCT plus nCRT group had a significantly higher proportion of patients who achieved a PCR (10.8% vs. 2.7%, *p* = 0.04) and who experienced downstaging (33.8% vs. 5.3%, *p* < 0.001). No significant differences between the two groups were observed in any of the other demographic or clinicopathological characteristics.

### 3.5 Survival outcomes after propensity score matching

#### 3.5.1 NCT alone group matched with nCRT alone group (3:1)

**FIGURE 4:**
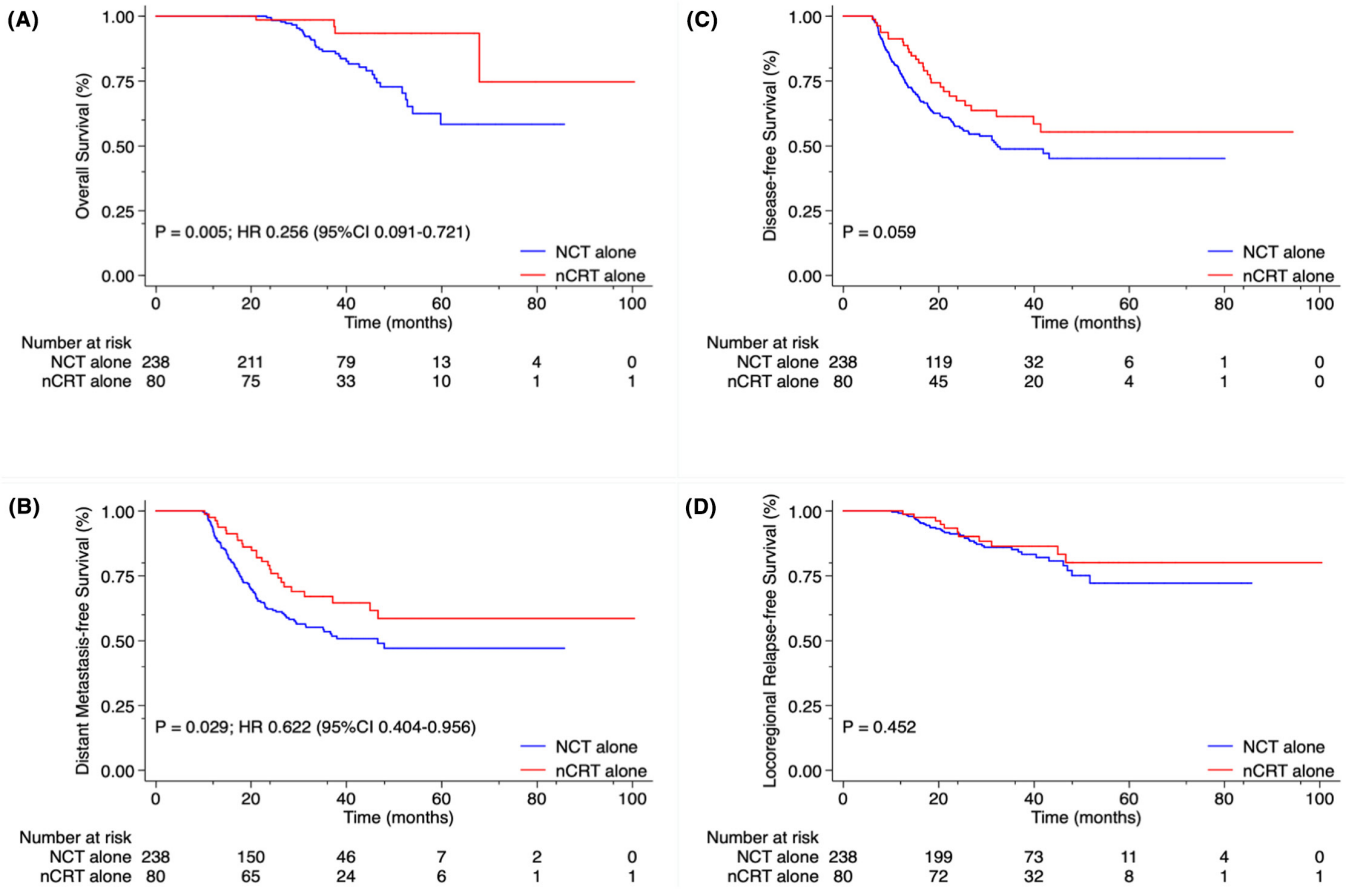
Kaplan–Meier curve analyses of patients with magnetic resonance imaging (MRI)‐detected extramural venous invasion (EMVI)‐positive locally advanced rectal cancer (LARC), comparing neoadjuvant chemotherapy (NCT) alone group (*N* = 238) with neoadjuvant chemoradiotherapy (nCRT) alone group (*N* = 80), after propensity score matching (3:1), for (A) overall survival, (B) distant metastasis‐free survival, (C) disease‐free survival, and (D) locoregional relapse‐free survival rates, January 2013 through October 2021.

After PSM was applied, the 3‐year OS rate (86.5%) and the 3‐year DMFS rate (53.5%) in the NCT alone group were both significantly lower than the 3‐year OS rate (98.6%) and the 3‐year DMFS rate (67.1%) in the nCRT alone group (HR = 3.903 and HR = 1.608; *p* = 0.005 and *p* = 0.03, respectively) (Figure 4A,B). The NCT alone group also demonstrated lower 3‐year DFS and LRFS rates, but these differences were not statistically significant (*p* = 0.06 and *p* = 0.45, respectively) (Figure 4C,D).

#### 3.5.2 nCRT alone group matched with NCT plus nCRT group (1:2)

**FIGURE 5:**
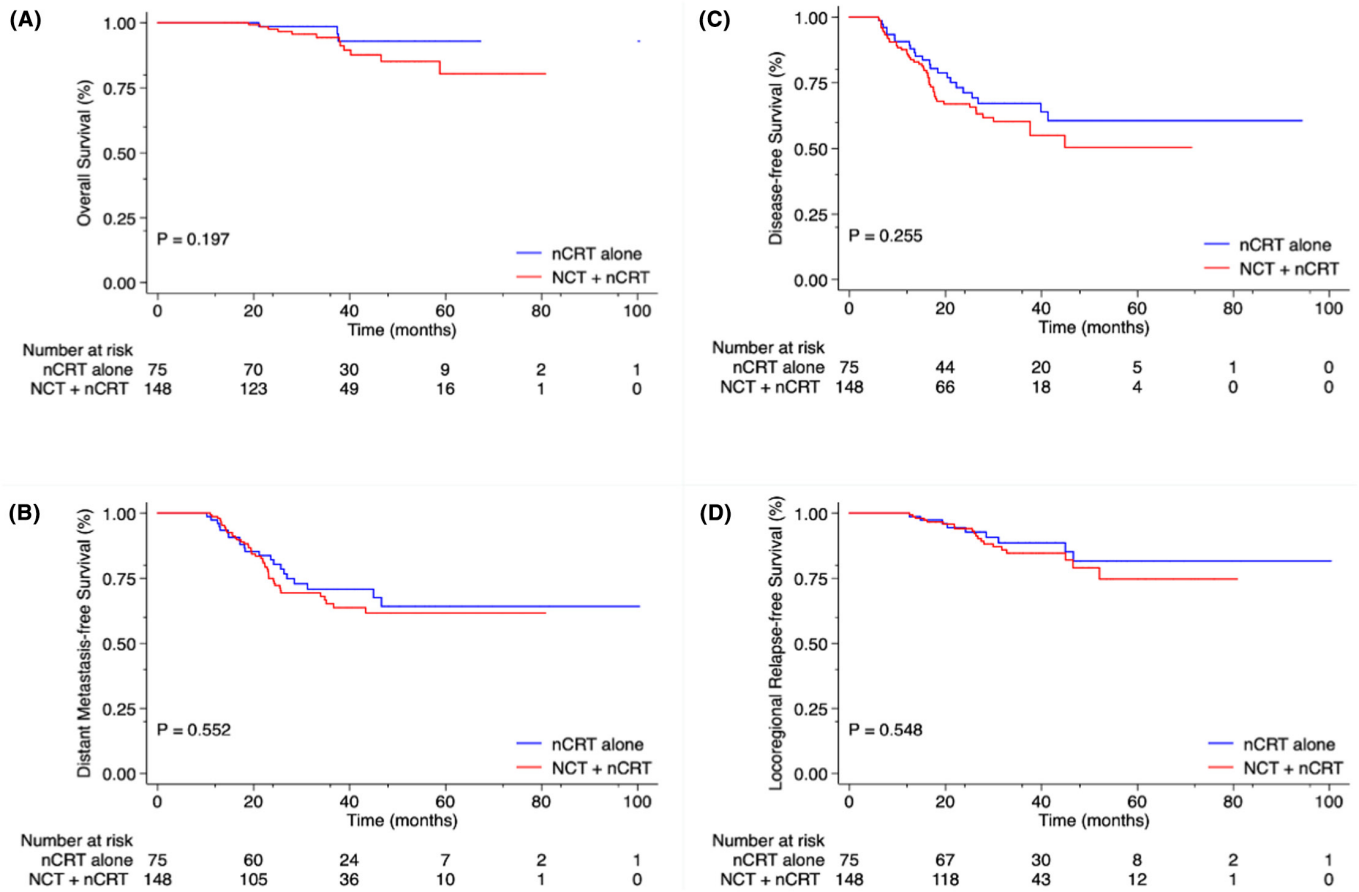
Kaplan–Meier curve analyses of patients with magnetic resonance imaging (MRI)‐detected extramural venous invasion (EMVI)‐positive locally advanced rectal cancer (LARC), comparing neoadjuvant chemoradiotherapy (nCRT) alone group (*N* = 75) with neoadjuvant chemotherapy (NCT) + nCRT group (*N* = 148), after propensity score matching (1:2), for (A) overall survival, (B) distant metastasis‐free survival, (C) disease‐free survival, and (D) locoregional relapse‐free survival rates, January 2013 through October 2021.

After PSM was applied, no statistically significant differences were observed in the 3‐year OS (98.5% vs. 94.3%; *p* = 0.20), DMFS (70.8% vs. 65.2%; *p* = 0.55), DFS (67.2% vs. 60.2%; *p* = 0.26); and LRFS (88.5% vs. 84.6%; *p* = 0.55) rates (Figure 5).

## 4 DISCUSSION

In this study, we investigated the efficacy of different neoadjuvant treatment regimens (with and without radiotherapy) for patients with rectal cancer who were mrEMVI‐positive at the time of diagnosis. We found that among LARC patients who were mrEMVI‐positive, those treated before surgery with nCRT experienced significantly higher 3‐year OS and DMFS rates than those treated with NCT. Also, we observed that the addition of NCT to nCRT prior to surgery resulted in improved PCR and downstaging rates, but without definite survival benefits.

Prior to the application of PSM, the 3‐year OS, DFS, DMFS, and LRFS rates in our study cohort were 90.4%, 57.5%, 61.1%, and 85.7%, respectively. These results were comparable to those from a pooled analysis of 33 articles involving mrEMVI‐positive patients with rectal cancer, the majority of who received nCRT prior to surgery; the authors reported 3‐year OS rates that ranged from 81.6% to 87.8%, and 3‐year DFS rates that ranged from 35% to 75%.^17^

Patients in our study who converted from a pre‐treatment mrEMVI‐positive status to a post‐treatment EMVI‐negative status on surgical pathology demonstrated significantly better 3‐year OS, DMFS, DFS, and LRFS rates than those who were EMVI‐positive on surgical pathology. Others have also observed that the resolution of EMVI after neoadjuvant treatment was associated with improved survival.^15^, ^17^, ^18^ Chand M, et al reported that not only did patients who were persistently EMVI‐positive have lower DFS rates than patients who became EMVI‐negative after neoadjuvant treatment, but also this was the case regardless of whether EMVI‐negative status was based on MRI after neoadjuvant treatment or histopathology after surgery.^20^

Patients in our study treated with NCT and no radiotherapy before surgery still had a high rate of EMVI conversion, which is consistent with what others have reported.^16^ However, the omission of radiotherapy in the neoadjuvant regimen did result in significantly unfavorable survival outcomes in our patients. Previous studies have explored the possibility of avoiding routine pelvic radiation by using NCT alone for rectal cancer, and they have reported lower toxicity rates than nCRT with comparable short‐term outcomes.^15^, ^16^, ^18^, ^21^ However, these studies have used a variety of different protocols, involved selection bias, and not been focused on mrEMVI‐positive patients. The latter is problematic for comparisons, because there is strong evidence that mrEMVI is an independent negative prognostic factor for oncologic outcomes in rectal cancer.^6^ One study with a somewhat more similar patient population, the GEMCAD 0801 prospective, phase II trial, involved 46 patients who received a multi‐drug NCT regimen prior to surgery in patients with high‐risk metastatic rectal cancer.^18^ This resulted in a 91% mrEMVI conversion rate among the 23 (50%) mrEMVI‐positive patients, but there was no survival improvement.

Furthermore, although an mrEMVI‐positive status has been shown to be associated less with local failure than with distant metastases, there are several reasons why NCT may still not be adequate treatment for these patients. First, an mrEMVI‐positive status is often accompanied by MRF involvement and LLN metastasis,^22^ both of which are known risk factors for local and distant recurrence. Second, there is a positive correlation between improved local control and decreased risk of distant relapse.^23^, ^24^ Third, while TME largely eliminates the potential of recurrence from lymphatic spread, it does not always interrupt the vascular pathways of spread that are beyond the mesorectal boundary.^18^ And fourth, the fact that the liver is the most common landing site of metastasis from rectal cancer suggests the particular importance of controlling that vascular pathway.^25^ All of these reasons suggest that pelvic radiotherapy, which can impede both local and regional spread of tumor cells,^17^ may play a pivotal role as part of neoadjuvant treatment for patients with mrEMVI‐positive LARC. Based on our results showing that radiotherapy may improve the survival outcomes of these patients, the omission of radiotherapy in these patients should be done with caution.

There is still no consensus on the optimal chemotherapy strategy for patients with LARC who are mrEMVI‐positive. An initial mrEMVI‐positive status correlates with a high risk of synchronous metastases and earlier distant failures,^1^, ^26^ both of which suggest the presence of micrometastases at the time of diagnosis. And, studies have demonstrated that when mrEMVI status has been converted from positive to negative, survival appears to improve and even revert back to EMVI‐negative levels.^17^, ^27^ Thus, the role of chemotherapy in the treatment strategy for these patients is likely crucial.

We did not find any significant differences in the survival rates of patients who received nCRT with or without NCT. However, a recent meta‐analysis showed that patients with LARC receiving NCT in conjunction with nCRT had better DFS and OS rates than those receiving nCRT alone,^28^ suggesting the importance of preoperative chemotherapy. In a trial involving patients with high‐risk LARC, of whom 30% were mrEMVI‐positive, those treated with a short‐course radiotherapy plus consolidation chemotherapy prior to surgery had a significantly lower probability of disease‐related treatment failure at 3 years than patients who treated with standard treatment regimen.^10^ The authors concluded that this was indicative of the increased efficacy of preoperative chemotherapy over ACT. Finally, others have reported that the addition of NCT before nCRT was significantly associated a high rate of mrEMVI conversion.^29^ Our results support that the addition of NCT to nCRT as a reasonable choice for mrEMVI‐positive patients with LARC, as this may address micrometastases, induce more mrEMVI conversions, and potentially improve oncologic outcomes.

In further support of the addition of NCT to nCRT, the patients who received this regimen did have significantly more probability of PCR and downstaging, when compared with patients in the group receiving nCRT alone prior to surgery. As noted above, the survival rates for these two groups of patients in our study did not differ significantly. The lack of a statistically significant difference may have been because a higher proportion of the patients in our NCT plus nCRT group had adverse prognostic features, including clinical T4 stage, MRF involvement, and LLN metastases. Although these differences also did not reach statistical significance in our study, these MRI‐defined factors are known to be associated with poor survival outcomes.^30^ In fact, Lord et al showed that using MRI‐based prognostic risk factors (e.g., EMVI, tumor deposits [TD], and MRF) resulted in better predictions of outcomes than more traditional TNM staging.^31^

Our results add to the growing evidence of the benefits of utilizing MRI‐based prognostic factors like mrEMVI status in the management of rectal cancer. Yet, many rectal cancer trials involving NCT are still using only traditional TNM staging for patient selection, stratification, and treatment decisions. For example, neither PRODIGE 23 nor FOWARC reported the EMVI status of patients on preoperative MRI.^11^, ^15^ In part because of this, there remains a lack of consensus regarding the ultimate impact of NCT on long‐term survival outcomes in patients with rectal cancer. Randomized trials that stratify patients by more comprehensive and precise prognostic factors are needed to clarify the clinical value of adding NCT to nCRT.

There are potential limitations of this analysis. Although PSM analysis was used to eliminate potential bias, the retrospective study design and relatively short follow‐up are limitations. Thus, our results should be interpreted with care. Further, we did not use the MRI‐based tumor regression grade scale for mrEMVI (mr‐vTRG) in our study. This would have been helpful to evaluate treatment responses, and it has been shown to be a useful tool to identify high‐risk patients after neoadjuvant treatment.^32^ Finally, although we reported on ACT received by patients, the study was focused on different neoadjuvant treatment regimens and their impact on postoperative EMVI status, so we did not include ACT in our analyses. It is possible that the exclusion of ACT from the analyses may have altered or biased some of our results.

## 5 CONCLUSION

In patients with LARC who were mrEMVI‐positive at the time of diagnosis, most converted from mrEMVI‐positive to mrEMVI‐negative after neoadjuvant treatment, and those who converted had better OS and DFS rates. Patients who received nCRT prior to surgery had more favorable survival outcomes than those who received NCT prior to surgery, suggesting the importance of including neoadjuvant radiotherapy in this high‐risk group. Patients who received NCT in addition to nCRT prior to surgery had higher rates of PCR and downstaging than those who had nCRT alone, but a difference in survival outcomes between the two groups was not identified. NCT plus nCRT may still be an appropriate option for patients with LARC who are mrEMVI‐positive. Results from larger, well‐designed prospective trials, using more comprehensive prognostic factors for patient selection, stratification, and treatment decisions are warranted in order to determine optimal neoadjuvant treatment strategies with the aim of further decreasing distant metastases and improving survival.

## AUTHOR CONTRIBUTIONS

**Mo Chen:** Investigation (equal); writing – original draft (equal). **Yan Ma:** Investigation (equal); supervision (equal); validation (equal). **Yi‐wen Song:** Data curation (equal); supervision (equal); validation (equal). **Jinhua Huang:** Data curation (equal); methodology (equal); supervision (equal); validation (equal). **Yuan‐hong Gao:** Methodology (equal); supervision (equal); validation (equal). **Jian Zheng:** Data curation (equal); funding acquisition (equal); supervision (equal). **Fang He:** Methodology (equal); supervision (equal); writing – review and editing (equal).

## FUNDING INFORMATION

This work was supported by the National Natural Science Foundation of China (No. 82171163); the program of Guangdong Provincial Clinical Research Center for Digestive Diseases (2020B1111170004).

## ETHICS STATEMENT

The experimental protocol was approved by the Central Ethics Committee of the Sixth Affiliated Hospital, Sun Yat‐sen University (No. 2022ZSLYEC‐624).

## Data Availability

The datasets used for this study are available from the corresponding author upon reasonable request.
